# Is urinary incontinence associated with sedentary behaviour in older women? Analysis of data from the National Health and Nutrition Examination Survey

**DOI:** 10.1371/journal.pone.0227195

**Published:** 2020-02-04

**Authors:** Javier Jerez-Roig, Joanne Booth, Dawn A. Skelton, Maria Giné-Garriga, Sebastien F. M. Chastin, Suzanne Hagen

**Affiliations:** 1 Research group on Methodology, Methods, Models and Outcomes of Health and Social Sciences (M_3_O). Faculty of Health Sciences and Welfare. Centre for Health and Social Care Research (CESS), University of Vic-Central University of Catalonia (UVic-UCC), Vic, Spain; 2 School of Health and Life Sciences, Glasgow Caledonian University, Glasgow, United Kingdom; 3 Blanquerna Faculty of Psychology, Education and Sport Sciences, Ramon Llull University, Barcelona, Spain; 4 Department of Sport and Movement Science, Ghent University, Ghent, Belgium; 5 Nursing, Midwifery and Allied Health Professions Research Unit, Glasgow Caledonian University, Glasgow, United Kingdom; University of Malaya, MALAYSIA

## Abstract

**Background:**

Urinary incontinence (UI) is a common geriatric syndrome associated with physical and cognitive impairments. The association between type of UI and sedentary behaviour (SB) has not been explored.

**Aim:**

To determine association between moderate-severe UI, or any stress UI (SUI) or any urgency UI (UUI) and SB in community-dwelling older women.

**Methods:**

Women aged 60 and over from the 2005–2006 cycle of the National Health and Nutrition Examination Survey (NHANES) with objectively measured (accelerometer) and self-reported SB and UI data were selected. Multivariate models exploring association between moderate-severe UI and SB, or SUI and SB, or UUI and SB were analysed using logistic regression adjusted for factors associated with UI.

**Results:**

In the overall sample of 459 older women, 23.5% reported moderate-severe UI, 50.5% reported any SUI and 41.4% reported any UUI. In bivariate analysis objectively measured proportion of time in SB was associated with moderate-severe UI and UUI (*p* = 0.014 and *p* = 0.047) but not SUI. Average duration of SB bouts in those with moderate-severe UI or any SUI was no longer than older women reporting no continence issues, but it was significantly (19%) longer in older women with any UUI (mean difference 3.2 minutes; *p* = 0.001). Self-reported SB variables were not associated with any type of UI. Multivariate analysis showed an association between UUI and a longer average duration of SB bouts (OR = 1.05, 95% CI = 1.01–1.09, *p* = 0.006) but no association with moderate-severe UI or SUI.

**Conclusion:**

UUI was significantly associated with increased average duration of SB bouts in community-dwelling older women. The importance of objective measurement of SB is highlighted and suggests that decreasing time in prolonged sitting may be a target intervention to reduce UUI. Future studies are required to further explore the association between SB and incontinence.

## Introduction

With the population ageing globally, the incidence of geriatric syndromes such as urinary incontinence (UI) is increasing [1]. This ‘frequently forgotten geriatric giant’ affects approximately 25% of the population worldwide and is particularly prevalent in older women, and individuals with cognitive, neurological and physical impairments [2–4]. UI is defined by the International Continence Society as the complaint of involuntary loss of urine and in women occurs as three main types: stress UI (SUI) with exercise, coughing, or sneezing; urgency UI (UUI) accompanied by a strong urge to urinate and mixed UI, which combines the two [5]. In the older population, physical activity (PA) plays an important role as a modifiable protective factor that can prevent or even reduce UI [6]. Several studies have analysed the relationship between UI and PA but most have used self-reported questionnaires to assess PA [4,7,8], which are known to overestimate actual PA when compared with objective measurement [9]. More recently, the independent effect of sedentary behaviour (SB) on poor health outcomes has emerged [10]. SB refers to activities that are performed in a sitting or reclining position and are low in energy expenditure (≤ 1.5 metabolic equivalents [METs]) [11].

Little evidence is available on any association between SB and UI. A recent literature review on SB and UI in women identified only five cross-sectional studies and concluded that SB may represent a risk factor for female UI [12]. However, data were limited to self-reported SB in four of the studies and no study analysing the association between SB and the different types of UI has been identified [12], i.e. SUI and UUI. The aetiology and risk factors for types of UI differ, thus the design of specific strategies to prevent and manage SUI and UUI needs to reflect evidence from analysis of associated factors for each separately [7]. The aim of this study was to evaluate the association between SB and moderate-severe UI, any SUI or any UUI in community-dwelling older women.

## Methods

### Study design and participants

We conducted an analysis of cross-sectional data from the 2005–2006 cycle of the National Health and Nutrition Examination Survey (NHANES). The NHANES consists of a series of complex and multi-stage surveys on a nationally representative, non-institutionalized U.S. population, conducted annually by the National Centre for Health Statistics, which is part of the Center for Disease Control and Prevention. Participants are selected randomly and provide data via a variety of self-reported questionnaires, physical and laboratory examinations. Ethics approval for NHANES was obtained by the National Center for Health Statistics Research Ethics Review Board [13]. Further information on the survey can be found on the NHANES website [14].

Women aged 60 and over were included in the analysis. Women with physical impairments preventing walking, incomplete UI questionnaire data and/or missing or invalid accelerometer data were excluded from the study. We aimed to achieve a final sample of at least 280, assuming a significance level of 0.05 and power of 0.80. This sample size calculation was based on a quantitative PA variable in the study conducted by Lee et al. (2012), since there are no studies comparing objective SB variables and UI types. This study verified a median of 693 and 792 METs min/week of total PA in older women with and without UI, respectively [15].

### Urinary incontinence variables

The study investigated three UI primary outcomes using 2005–2006 NHANES data. Moderate-severe UI (of any type), corresponded to ‘at least weekly leakage, or monthly leakage of volumes more than just drops’. The dichotomous variable was calculated from the severity index, a product of the frequency of UI episodes (1. < once a month, 2. a few times a month, 3. a few times a week or 4. every day and/or night) and amount of leakage (1. drops, 2. splashes or 3. more) [16,17]. Severity scores ranged from 1 to 12 (mild symptoms 1–2, moderate symptoms 3–6, severe symptoms 7–9, very severe 10–12) and scores of 3 or over were categorised as moderate-severe UI. Those who reported that during the past 12 months they leaked or lost control of even a small amount of urine with an activity like coughing, lifting or exercise were considered to have SUI. Participants answering yes to the NHANES question “during the past 12 months have you leaked or lost control of even a small amount of urine with an urge or pressure to urinate and couldn’t get to the toilet fast enough?” were considered to have UUI. These three UI variables (moderate-severe UI, SUI, UUI) were the dependent variables in our separate analyses.

### Sedentary behaviour variables

SB variables included self-reported and objectively measured information. The objective measures were collected by a physical activity monitor (PAM), which was worn on the hip for seven consecutive days during waking hours. The PAM model used in the cycle of 2005–2006 of NHANES was the accelerometer Actigraph 7164 (Actigraph, LLC, Fort Walton beach, FLA). Accelerometry data were considered valid if at least 5 days with 10 hours of continuous wear time were available. The PAM sums acceleration counts over a 1-minute epoch. Epochs with < 100 counts per minute (cpm) were classified as SB [18]. For each individual the proportion of daily waking time spent in SB (% time in SB) variable was computed by summing the number of epochs classed as SB and dividing by the total wear time. This was then averaged over the valid days for each individual. In addition, for each individual the average length of the SB bouts (average SB bout length) in minutes was computed using the methods developed by Chastin *et al*. [19,20]. As data on the length of SB bout is not normally distributed, averaging cannot be performed using standard arithmetic mean or median.

Self-reported SB included number of hours per day sitting watching TV/videos over the past 30 days, and an estimate of SB in usual daily activities (e.g. work-related, household chores). Both variables were recoded as 4-category and 3-category ordinal variables, according to the distribution (see Table 1). All these SB variables were independent variables in our main analyses.

**Table 1:** Descriptive analysis of categorical variables for older women with accelerometry data (n = 459) from the NHANES study (2005–2006).

Variables	n	%
Age		
60–64	122	26.6
65–69	104	22.7
70–74	86	18.7
75–79	49	10.7
80–84	61	13.3
≥85	37	8.1
Race		
White non-Hispanic	285	62.1
Black non-Hispanic	84	18.3
Hispanic	79	17.2
Others (Asian, etc)	11	2.4
BMI		
Eutrophic (18.5–24.9 kg/m^2^)	152	33.1
Overweight (25.0–29.9 kg/m^2^)	141	30.7
Obese (30.0–39.9 kg/m^2^)	133	29.0
Extreme obese (≥40.0 kg/m^2^)	30	6.5
Conditions		
Arthritis	269	58.6
Thyroid problem	122	26.6
Malignancy	97	21.1
Diabetes	96	20.9
Asthma	52	11.3
Chronic Bronchitis	42	9.2
Stroke	31	6.8
Congestive heart failure	29	6.3
Angina pectoris	28	6.1
Coronary heart disease	26	5.7
Heart attack	25	5.4
Emphysema	21	4.6
Liver condition	18	3.9
Smoking habit		
No	266	58.0
Yes (former or current)	193	42.0
Continence status (urinary)		
Dry	257	56.0
Slight incontinence	94	20.5
Moderate incontinence	69	15.0
Severe incontinence	39	8.5
Self-reported SB in daily activities		
Sitting and not walking very much	117	25.5
Standing or walking quite a lot	273	59.5
Heavy work, lifting/carrying loads or climbing	69	15.0
Self-reported watching TV/videos		
< 1 hour/day	50	10.9
1–2 hours/day	172	37.5
3–4 hours/day	141	30.7
≥ 5 hours/day	93	20.3

### Other variables

Other independent variables known to be associated with UI in older women and therefore included in the analyses were: age (categorised as an ordinal variable by 5-year categories), race, body mass index (BMI) calculated as kg/m^2^, number of vaginal deliveries, smoking habit (never versus current/former smoker), number of alcoholic drinks per week and number of comorbidities. Race was categorised as non-Hispanic white, non-Hispanic black, Hispanic (including Mexican American), and others (including multi-racial), according to previous studies [15,16]. Data from women in the “others” category was included for the descriptive analysis but not included in the multivariate analysis due to the low number of cases.

### Statistical analysis

Descriptive analysis was performed for all dependent and independent variables presenting absolute and relative frequencies for categorical variables and mean and standard deviation (SD) for continuous variables. In the bivariate analysis, Chi-square test was applied for the dichotomised smoking variable, the Chi-square for linear trend test for age and race variables and Student-t test for all continuous variables. Multivariate analysis was carried out separately for the three dependent variables of moderate-severe UI, any SUI and any UUI to evaluate the associated factors for each type of UI. For this purpose, logistic regression using the stepwise method was employed. Permanence of variables in multiple analysis depended on the statistical significance of the covariates, the likelihood ratio test, absence of multi-collinearity, and on the capacity for improving the model through the goodness of fit test for logistic regression (Hosmer-Lemeshow), which was used to check the fit of the final models. The magnitude of association was determined by the odds ratio (OR), and 95% confidence interval for categorical variables and by the mean difference for continuous variables. Associations were considered statistically significant if *p*<0.05.

## Results

### Characteristics of participants

**Fig 1:**
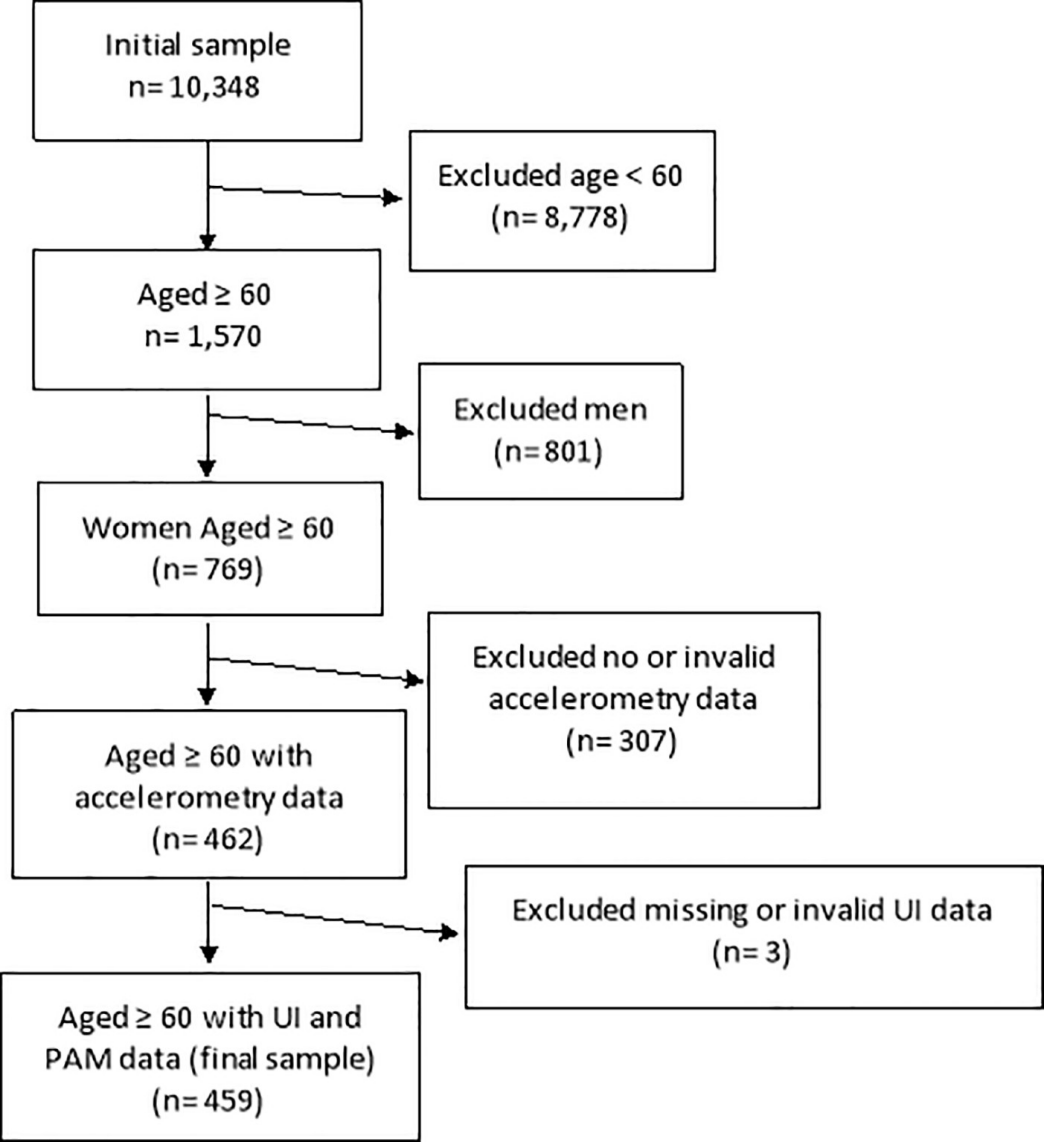
Sample selection flow-chart of community-dwelling older women from the NHANES study (2005–2006).

The NHANES database of cycle 2005–2006 contains 10,348 participants. Of these, 8,778 were excluded because they were younger than 60 years of age and 801 men were excluded. Another 307 were excluded because there was no valid accelerometry data and a further 3 for not completing the urinary incontinence questions. There were no differences between the women aged 60 and over who were included in the study and those who were not, with respect to race/ethnicity, BMI, smoking and medical conditions, other than stroke where there were 6.8% in the included group versus 12.9% in those women excluded (*p* = 0.004). Included women were significantly younger than excluded, 71.0 (SD: 7.98) versus 73.95 (8.77) respectively, according to the Mann-Whitney test (*p*<0.001). The final sample consisted of 459 community-dwelling older women (see Fig 1), whose main characteristics are described in Table 1 and Table 2. Overall, these older women spent 64% (SD: 12.2) of their waking time sitting or lying and were sedentary for an average bout duration of 16.8 minutes. 23.5% (n = 108) reported moderate-severe UI; 232 (50.5%) reported SUI and 190 (41.4%) reported UUI.

**Table 2:** Descriptive analysis of continuous variables for older women with accelerometry data (n = 459) from the NHANES study (2005–2006).

Variables	Mean	Standard deviation
Number of comorbidities	2.5	1.8
Average SB bout length (minutes)	16.8	8.7
% time in SB	64.0	12.2
Number of alcoholic drinks/week	1.2	3.3
Number of vaginal deliveries	3.6	2.6

A statistically significant difference was found in reported SUI among women of different races (see Table 3), where prevalence was higher in Hispanic women and white, non-Hispanic women than black, non-Hispanic women.

**Table 3:** Bivariate analysis of the association between type of UI reported by all older women with accelerometry data and categorical variables (age, race and smoking habit).

	Moderate-severe UI		Any SUI		Any UUI	
	Cases (%)	*p-value*	Cases (%)	*p-value*	Cases (%)	*p-value*
Age^a^						
60–64	26 (21.3%)	0.072	64 (52.5%)	0.372	43 (35.2%)	0.300
65–69	22 (21.2%)		57 (55.3%)		49 (47.1%)	
70–74	17 (19.8%)		42 (48.8%)		36 (42.4%)	
75–79	10 (20.4%)		21 (42.9%)		18 (36.7%)	
80–84	24 (39.3%)		30 (49.2%)		26 (42.6%)	
≥85	9 (24.3%)		18 (50.0%)		18 (50.0%)	
Race^a^						
Hispanic	22 (27.8%)	0.092	46 (58.2%)	0.005*	34 (43.0%)	0.862
White non-Hispanic	71 (24.9%)		154 (54.2%)		118 (41.7%)	
Black non-Hispanic	14 (16.7%)		30 (36.1%)		35 (41.7%)	
Smoke (ever)^b^	38 (19.7%)	0.098	96 (50.0%)	0.780	71 (37.0%)	0.090

a Chi-square (for linear-trend) test

b Chi-square

* Statistically significant (*p*<0.05)

Table 4 presents the bivariate analyses between older women with accelerometry data who reported moderate-severe UI, any SUI, any UUI *and* continuous variables; the comparison groups consist of all older women without the above mentioned conditions. The mean differences in the accelerometer-derived variable % time in SB show that older women with UI of all types were sedentary for a greater proportion of time than those without UI and the association was significant for moderate-severe UI and UUI. For older women with any UUI the average duration of bouts of SB was 19% longer (3.23 min per bout, *p* = 0.001) than the average duration of SB bouts for women in the sample without the condition, including those with no UI and those with other types of UI. The accelerometer-derived SB variable ‘% time in SB’ was significantly associated with moderate-severe UI and UUI and ‘average duration SB bouts’ was significantly associated with UUI, in contrast to the self-reported SB variables where none were significantly associated with UI. BMI was significantly higher in those with SUI and UUI, and number of comorbidities was also significantly higher in those with moderate-severe UI and SUI.

**Table 4:** Bivariate analyses of the association between moderate-severe UI, any SUI and any UUI *vs* continuous independent variables among older women with accelerometry data.

	Moderate-severe UI		Any SUI		Any UUI	
Independent variables	Mean difference	*p-value*	Mean Difference	*p-value*	Mean difference	*p-value*
Objective SB variables						
% time in SB	3.268	0.014*	0.787	0.488	2.295	0.047*
Average SB bouts duration (min)	1.836	0.086	0.512	0.538	3.231	0.001*
Self-reported SB variables:						
Daily activities	0.045	0.517	-0.008	0.898	0.076	0.202
Hours watching TV/videos	0.295	0.094	0.095	0.526	0.166	0.276
BMI (kg/m^2^)	1.188	0.103	2.258	<0.001*	2.024	0.001*
Vaginal deliveries	0.234	0.414	0.306	0.212	0.406	0.112
Drinks/week	0.116	0.747	0.278	0.363	-0.219	0.480
Number of comorbidities	0.407	0.034*	0.427	0.009*	0.179	0.281

* Statistically significant (*p*<0.05)

The main results of the multivariate analysis are shown in Table 5; separate comparisons between women with and without the UI conditions were made, i.e. moderate-severe UI, as well as any SUI or any UUI. All three models were adjusted for age, number of comorbidities and number of vaginal deliveries. In addition, BMI was positively and significantly associated with UI in all models. Black non-Hispanic race was negatively and significantly associated with SUI. There was a significant association between longer ‘mean duration of SB bouts’ and reporting UUI. The results of the Hosmer-Lemeshow test were 0.491, 0.903 and 0.399 for moderate-severe UI, SUI and UUI models, respectively.

**Table 5:** Multivariate analysis models between moderate-severe UI, any SUI and any UUI and % time in SB and other independent variables in older women with accelerometry data.

	Model for moderate-severe UI (n = 422)		Model for any SUI (n = 420)		Model for any UUI (n = 420)	
Independent variables	OR (CI: 95%)	*p-value*	OR (CI: 95%)	*p-value*	OR (CI: 95%)	*p-value*
% time in SB	3.69 (0.38–35.98)	0.140	1.76 (0.25–12.18)	0.569	3.04 (0.44–21.10)	0.261
Age						
60–64	Reference	0.334	Reference	0.515	Reference	0.346
65–69	0.79 (0.40–1.54)	0.483	0.92 (0.51–1.64)	0.772	1.43 (0.81–2.53)	0.217
70–74	0.72 (0.35–1.51)	0.387	0.62 (0.33–1.15)	0.129	1.19 (0.64–2.22)	0.583
75–79	0.79 (0.33–1.91)	0.602	0.53 (0.25–1.13)	0.099	0.98 (0.46–2.11)	0.996
80–84	1.70 (0.78–3.69)	0.181	0.65 (0.31–1.36)	0.256	1.19 (0.58–4.47)	0.634
≥85	0.96 (0.36–2.61)	0.942	0.74 (0.30–1.81)	0.504	2.55 (1.04–6.28)	0.041*
BMI	1.04 (0.99–1.07)	0.070	1.06 (1.02–1.10)	0.001*	1.06 (1.02–1.09)	0.002*
Race						
Hispanic	Reference	0.061	Reference	<0.001*	Reference	0.618
White non-Hispanic	0.71 (0.37–1.35)	0.291	0.88 (0.49–1.57)	0.662	1.02 (0.57–1.80)	0.957
Black non-Hispanic	0.36 (0.16–0.85)	0.019*	0.27 (0.13–0.54)	<0.001*	0.77 (0.39–1.54)	0.461
Number of comorbidities	1.07 (0.93–1.23)	0.381	1.13 (0.99–1.29)	0.056	0.94 (0.83–1.07)	0.332
Number of vaginal deliveries	1.04 (0.95–1.14)	0.391	1.05 (0.97–1.14)	0.255	1.08 (0.99–1.17)	0.072

* Statistically significant (<0.05)

The proportion of time in SB and the self-reported SB variables showed no significant association with any type of UI in the multivariate analyses (see Table 6, Table 7 and Table 8).

**Table 6:** Multivariate logistic regression models regressing moderate-severe UI, any SUI and any UUI on average duration of SB bouts and other independent variables in older women with accelerometry data.

	Model for moderate-severe UI (n = 329)		Model for any SUI (n = 327)		Model for any UUI (n = 327)	
Independent variables	OR (CI: 95%)	*p-value*	OR (CI: 95%)	*p-value*	OR (CI: 95%)	*p-value*
Average duration of SB bouts (min)	1.01 (0.98–1.05)	0.360	1.01 (0.98–1.04)	0.641	1.05 (1.01–1.09)	0.006*
Age						
60–64	Reference	0.306	Reference	0.401	Reference	0.361
65–69	0.65 (0.30–1.42)	0.277	0.89 (0.44–1.79)	0.738	1.44 (0.72–2.89)	0.304
70–74	0.43 (0.18–1.05)	0.063	0.50 (0.24–1.05)	0.067	1.22 (0.59–2.53)	0.599
75–79	0.78 (0.31–1.99)	0.609	0.52 (0.22–1.21)	0.129	0.99 (0.42–2.33)	0.982
80–84	1.20 (0.51–2.85)	0.677	0.55 (0.24–1.26)	0.157	0.85 (0.37–1.95)	0.694
≥85	0.91 (0.32–2.54)	0.851	0.65 (0.25–1.68)	0.373	2.34 (0.89–6.11)	0.084
BMI	1.06 (1.01–1.10)	0.018*	1.07 (1.02–1.11)	0.003*	1.06 (1.02–1.10)	0.008*
Race						
Hispanic	Reference	0.147	Reference	<0.001*	Reference	0.573
White non-Hispanic	0.94 (0.42–2.11)	0.879	0.95 (0.46–1.96)	0.880	0.90 (0.38–2.13)	0.809
Black non-Hispanic	0.44 (0.16–1.20)	0.109	0.25 (0.11–0.61)	0.002*	1.25 (0.59–2.61)	0.561
Number of comorbidities	0.98 (0.84–1.15)	0.791	1.13 (0.98–1.30)	0.100	0.92 (0.80–1.06)	0.232
Number of vaginal deliveries	1.06 (0.96–1.18)	0.224	1.05 (0.96–1.15)	0.299	1.09 (0.99–1.20)	0.066

* Statistically significant (<0.05)

**Table 7:** Multivariate analysis models between moderate-severe UI, any SUI and any UUI and self-reported SB in daily activities and other independent variables in community-dwelling older women.

	Model for moderate-severe UI (n = 422)		Model for SUI (n = 420)		Model for UUI (n = 420)	
Independent variables	OR (CI: 95%)	*p-value*	OR (CI: 95%)	*p-value*	OR (CI: 95%)	*p-value*
SB (daily activities)	0.93 (0.63–1.37)	0.699	0.88 (0.63–1.24)	0.468	1.02 (0.73–1.43)	0.900
Age						
60–64	Reference	0.224	Reference	0.564	Reference	0.286
65–69	0.79 (0.40–1.56)	0.501	0.93 (0.52–1.67)	0.820	1.43 (0.81–2.52)	0.223
70–74	0.75 (0.36–1.56)	0.444	0.63 (0.34–1.17)	0.143	1.23 (0.66–2.28)	0.518
75–79	0.86 (0.36–2.06)	0.728	0.56 (0.26–1.19)	0.129	1.04 (0.49–2.21)	0.928
80–84	1.90 (0.89–4.06)	0.098	0.70 (0.34–1.43)	0.325	1.30 (0.64–2.65)	0.468
≥85	1.14 (0.42–3.06)	0.792	0.82 (0.34–2.00)	0.667	2.82 (1.16–6.87)	0.022*
BMI	1.04 (1.00–1.08)	0.044*	1.07 (1.03–1.10)	0.001*	1.06 (1.02–1.10)	0.001*
Race						
Hispanic	Reference	0.088	Reference	<0.001*	Reference	0.651
White non-Hispanic	0.75 (0.40–1.41)	0.373	0.89 (0.50–1.58)	0.686	1.08 (0.61–1.90)	0.804
Black non-Hispanic	0.40 (0.17–0.91)	0.029*	0.28 (0.14–0.56)	<0.001*	0.82 (0.42–1.63)	0.574
Number of comorbidities	1.09 (0.95–1.25)	0.211	1.15 (1.02–1.31)	0.028*	0.96 (0.84–1.08)	0.471
Number of vaginal deliveries	1.04 (0.95–1.14)	0.407	1.05 (0.97–1.14)	0.263	1.08 (0.99–1.16)	0.077

* Statistically significant (<0.05)

**Table 8:** Multivariate analysis models between moderate-severe UI, any SUI and any UUI and ‘hours watching TV/videos’ and other independent variables in community-dwelling older women.

	Model for moderate-severe UI (n = 420)		Model for SUI (n = 418)		Model for UUI (n = 418)	
Independent variables	OR (CI: 95%)	*p-value*	OR (CI: 95%)	*p-value*	OR (CI: 95%)	*p-value*
Hours watching TV/videos	1.06 (0.91–1.23)	0.479	1.00 (0.88–1.15)	0.970	1.04 (0.91–1.19)	0.533
Age						
60–64	Reference	0.357	Reference	0.563	Reference	0.246
65–69	0.76 (0.39–1.50)	0.435	0.93 (0.52–1.66)	0.798	1.46 (0.82–2.58)	0.196
70–74	0.73 (0.35–1.52)	0.396	0.63 (0.34–1.18)	0.152	1.26 (0.68–2.34)	0.468
75–79	0.82 (0.34–1.97)	0.655	0.55 (0.26–1.17)	0.119	1.06 (0.50–2.27)	0.874
80–84	1.68 (0.77–3.64)	0.192	0.67 (0.33–1.40)	0.288	1.25 (0.60–2.57)	0.553
≥85	1.07 (0.40–2.81)	0.897	0.79 (0.33–1.90)	0.596	2.91 (1.21–7.02)	0.018*
BMI	1.04 (0.99–1.08)	0.065	1.06 (1.02–1.10)	0.001*	1.06 (1.02–1.09)	0.002*
Race						
Hispanic	Reference	0.075	Reference	<0.001*	Reference	0.684
White non-Hispanic	0.76 (0.41–1.43)	0.397	0.90 (0.51–1.59)	0.709	1.05 (0.60–1.86)	0.856
Black non-Hispanic	0.39 (0.17–0.89)	0.025*	0.28 (0.14–0.56)	<0.001*	0.82 (0.42–1.62)	0.571
Number of comorbidities	1.08 (0.94–1.24)	0.298	1.14 (1.01–1.30)	0.042*	0.95 (0.83–1.07)	0.376
Number of vaginal deliveries	1.04 (0.95–1.14)	0.397	1.05 (0.97–1.14)	0.260	1.08 (0.99–1.17)	0.076

* Statistically significant (<0.05)

## Discussion

This cross-sectional study using NHANES data, aimed to explore the relationship between the severity of any UI and SB in community-dwelling older women. It also sought to separately explore the relationship between SUI and SB and UUI and SB in this population. Our results indicate that SB was not associated with increased risk of moderate to severe UI overall, or risk of SUI but they show the average duration of sedentary bouts is significantly associated with UUI. Being sedentary for 19% (3.23 minutes) longer per bout, on average, was significantly associated with reported UUI in older women. This is the first time this association has been shown using objectively measured SB. The results highlight the importance of distinguishing the type of UI in identifying specific risk factors for the complex condition of UI. While acknowledging the cross sectional nature of the study and therefore the inability to show direction of association these findings provide objective support for the first time, of the causal pathway between low physical activity and development of UUI/overactive bladder (OAB) demonstrated by McGrother et al (2012), using self-reported Leicester study data. They are also in line with Virtuoso et al. (2011) findings of urgency symptoms in older women who self-reported SB using a standardised questionnaire [21,22].

Our finding that SB is associated with UUI may be interpreted in relation to the pathophysiology of UUI, which differs from the biomechanical aetiology of SUI. UUI is complex and while aetiology is not fully understood, it is known to be associated with metabolic syndrome (MetS) [23]. SB is an independent risk factor for development of MetS, along with poor eating habits and obesity [24]. Risk of UUI is trebled in obese women, with and without diabetes [25] and peri-menopausal hormonal changes lead to increased total and abdominal fat in women, thus further increasing risk of MetS [26]. In this study BMI was a significant factor in all models of UI, providing support for the increased risk for UUI observed. Although it has not been fully elucidated, the effects of MetS on the bladder may occur through impact on the metabolically-active urothelium [27], which may be compromised by direct inflammatory effects on the autonomic nervous supply [28], via atherosclerosis-induced ischemia [29] or a combination of both mechanisms [23].

A further consideration adding to the possible explanations is the recently observed association between frailty and UUI, whereby frailty significantly predicts overactive bladder, but age does not [30]. No association between frailty and SUI in women has been found. Sedentary behaviour is associated with the development of frailty and a similar inflammatory-mechanism is proposed involving obesity and the MetS [31]. It is plausible that these factors of SB, frailty and UUI are linked in a common pathway based on inflammatory processes, however the detailed mechanisms have yet to be elicited. Alternatively, a simpler explanation for older women with UUI engaging in prolonged sitting could be that UUI is generally more unpredictable and distressing than SUI, and for these reasons they are more reluctant to move around.

The association between SB and UUI has potentially been seen in this study because SB was objectively measured. The literature shows that for SB, self-reported information using questionnaires presents limitations including under-reporting by at least 2 hours [29], reporting bias attributed to the need to provide socially desirable responses and the fact that estimating frequency and duration of SB is cognitively challenging for older adults [32]. Our use of objectively measured SB is a strength of this study and confirms the importance of using such approaches as the self-reported SB data showed no association with any type of UI in the analysis.

Several potential confounding factors have been identified in the literature, such as age, BMI or comorbidities [12]. In line with the literature, BMI is an important associated factor that was significant in all three models (for moderate-severe, stress and urgency UI) [33–35], although the mechanism by which it exerts its effects is likely to differ by type of UI. As suggested above raised BMI may directly contribute to UUI through inflammatory mechanisms.

Strengths of the study are the robustness of the population-based NHANES database and the range and specificity of variables to assess incontinence and SB (self-reported and objective). We controlled for several potential confounders in the analysis including age, BMI and number of vaginal deliveries. We note the significantly older age of the women aged 60 and over who were excluded compared to our sample and suggest that given the increased prevalence of UUI with ageing we would likely find similar or stronger association between UUI and SB if accelerometry data were available for this group.

Study limitations included a relatively small sample size and missing data. During the sample selection process, we needed to exclude a number of individuals due to missing values in the UI and/or objective SB variables. Most variables had missing data proportions under 5%, however ‘average duration of SB bouts’ was found to have 104 (22.7%) missing values. Nevertheless, we achieved an appropriate sample size selected from a large population-based study that is representative of the US population. The questions used in the NHANES survey are a potential limitation as they are specific to NHANES and are not validated questions recognised by the International Continence Society as most effective for identifying UI, severity of UI and type of UI. A further issue is the cross-sectional study design which cannot establish any linear (cause-effect) relationship between the two variables.

The analysis of accelerometer-measured data on SB from the NHANES national dataset showed objectively for the first time that SB and UUI are associated and that sedentary bouts in older women with UUI are on average almost a fifth longer than those without UUI. The results indicate that attention should be paid to establishing whether reducing sedentary time has the potential to improve UUI in older women. Such future research may help to clarify the association between these two important aspects of older women’s health and enable effective strategies to maintain or enhance continence to be developed.
